# Characterization of Scintillating X-ray Optical Fiber Sensors

**DOI:** 10.3390/s140203445

**Published:** 2014-02-19

**Authors:** Dan Sporea, Laura Mihai, Ion Vâţă, Denis McCarthy, Sinead O'Keeffe, Elfed Lewis

**Affiliations:** 1 National Institute Laser, Plasma and Radiation Physics, 409 Atomiştilor St., Măgurele RO-077125, Romania; E-Mail: laura.mihai@inflpr.ro; 2 “Horia Hulubei” National Institute of Physics and Nuclear Engineering, 30 Reactorului St., Măgurele RO-077125, Romania; E-Mail: vata@nipne.ro; 3 Optical Fibre Sensors Research Centre, ECE Department, University of Limerick, Limerick, Ireland; E-Mails: denis.mccarthy@emc.com (D.M.); Sinead.OKeeffe@ul.ie (S.O.); Elfed.Lewis@ul.ie (E.L.)

**Keywords:** fiber optic sensor, radiation monitoring in radiotherapy and industry, personnel dosimetry, radioluminescence, X-ray detector, X-ray fluorescence, X-ray tomography

## Abstract

The paper presents a set of tests carried out in order to evaluate the design characteristics and the operating performance of a set of six X-ray extrinsic optical fiber sensors. The extrinsic sensor we developed is intended to be used as a low energy X-ray detector for monitoring radiation levels in radiotherapy, industrial applications and for personnel dosimetry. The reproducibility of the manufacturing process and the characteristics of the sensors were assessed. The sensors dynamic range, linearity, sensitivity, and reproducibility are evaluated through radioluminescence measurements, X-ray fluorescence and X-ray imaging investigations. Their response to the operating conditions of the excitation source was estimated. The effect of the sensors design and implementation, on the collecting efficiency of the radioluminescence signal was measured. The study indicated that the sensors are efficient only in the first 5 mm of the tip, and that a reflective coating can improve their response. Additional tests were done to investigate the concentricity of the sensors tip against the core of the optical fiber guiding the optical signal. The influence of the active material concentration on the sensor response to X-ray was studied. The tests were carried out by measuring the radioluminescence signal with an optical fiber spectrometer and with a Multi-Pixel Photon Counter.

## 1. Introduction

Fiber optic sensors designed for radiation dosimetry, detection or monitoring fall into two categories: intrinsic sensors and extrinsic sensors [1]. In the case of intrinsic sensors the optical fiber constitutes both the sensing medium and the propagation one. Effects such as the radiation induced luminescence (RIL), the radiation induced absorption (RIA) [2], the generation of Cerenkov radiation in silica or plastic optical fibers exposed to charged particles [3,4], the change of the silica density [5,6] or the modification of the refractive index of silica under irradiation [7] were used to detect ionizing radiations. In extrinsic optical fiber sensors, the optical fiber is used only to transfer the optical signal from the radiation sensitive head towards the optical detector. These sensors are based on: thermoluminescence [8], optically stimulated luminescence [9,10] and scintillation (employing organic or inorganic materials) [11–13].

A novel technique to produce an extrinsic, scintillation type, optical fiber X-ray sensor is briefly introduced. The sensor is intended to be used as a low energy X-ray detector for monitoring radiation levels in radiotherapy environments, industrial X-ray applications and for personnel dosimetry. This paper presents the use of a combination of X-ray fluorescence, radioluminescence and X-ray imaging tests to evaluate the sensors characteristics and to assess the influence of the active material concentration and uniformity on the sensor response to X-ray. In addition, an evaluation of the sensors responsivity to the effects of two optically reflecting materials was studied.

## 2. Materials and Experiment

The sensor consists of a 550 mm PMMA type FDPF 4001 EH optical fiber, having a fiber/jacket diameter of 1 mm/2.2 mm, and a polyethylene jacket. The jacket was striped at both ends over a length of 10 mm. A SMA 950 connector was mounted at one end, whereas the sensor tip is located at the other end. The sensor investigated in the present study embeds the Gd_2_O_2_S:Tb phosphor [14]. The sensor was manufactured by preparing a mixture of phosphorous scintillating material with an epoxy solution produced by Struers (Catcliffe Rotherham, S60 5BL, UK) and known as “Epofix” [15]. Six sensors were produced with the same technology.

Samples 55 to 58 were produced using a plastic cylinder type mould of 3 mm internal diameter, while sensors 59 and 60 were developed using a heat shrink type mould of 2 mm internal diameter. Sensors 55, 56, and 59 have the length of 10 mm, while for sensors 57, 58, and 60 the length is 5 mm. The diameter of sensors 55 to 58 is 3 mm, and the diameter of sensors 59 and 60 is 2 mm. All sample fiber cores were prepared with the same metallurgical sanding and polishing methods [15]. The sensors' performances and quality were tested by combining X-ray fluorescence, radioluminescence, and X-ray imaging tests carried out on the sensor tip.

The purpose of the reported investigations was to evaluate six different sensors, prepared with the same technology, in order: to identify the active constituents concentration in the sensor tip; to evaluate the spatial distribution of the phosphor material constituents (Gd and Tb) in each sensor tip; to assess the concentricity of the sensor tip against the optical fiber core. The reproducibility of the sensors response to X-ray was also estimated, along with the spatial distribution of this responsivity along the tip length. The sensors response to various operating conditions of the X-ray source was also assessed. In addition, the effect produced by two reflecting materials covering the sensor tip on its response is reported.

The set-up for X-ray fluorescence investigations consist of a miniature AMPTEK Inc. X-ray tube system (AMPTEK INC., Bedford, MA, USA), operating with an Ag target [16]. The generated X-ray beam was focused, at about 2 mm spot diameter, on the optical fiber sensor tip. The fluorescence signal was picked up by a Silicon Drift Detector [17]. The entire system is controlled via USB connection by the Mini-X Control Software, making possible to set the voltage and current of the source, and the XRF-FP Quantitative Analysis Software for data analysis. The tube voltage was varied from 10 to 40 kV, while the current was modified from 5 to 90 µA. The dose rate at 40 kV/50 µA driving conditions applied to the source, at the sample distance of 30 cm is approximately 800 μGy/h, the on-axis flux value being 1.3 × 10^6^ cps/mm^2^ (40 cm working distance, at 40 keV/100 μA). The fluorescence detecting system accommodates a 25 mm^2^ sensitive area, with an energy resolution of 125–140 eV FWHM at 5.9 keV (55Fe), peak-to-background ratio of 20,000:1, and background counts <3 × 10^−3^/s, for 2 to 150 keV. For the fluorescence measurements, the X-ray source was operated at 30 kVp voltage and 10 μA current. During the entire study we kept the same excitation-detection geometry.

The radioluminescence was generated in the sensor tip by exposing it to the X-ray beam from the miniature AMPTEK Inc. X-ray tube system. The signal produced in the scintillating material, coupled to the plastic optical fiber, was detected by a scientific grade QE65000 optical fiber spectrometer (Ocean Optics, Dunedin, FL, USA) [18]. The operating parameters of the spectrometer for this application were: integration time 30 s and average 1. During the radioluminescence tests the sensor was kept in dark, obtaining a dark count of 2,500 counts. The incident angle of the X-ray beam used for radioluminescence measurements with the normal to the sensor surface was 22.5 degree.

Alternatively, the signal was also monitored by a type C10507-11-100U Multi-Pixel Photon Counter (MPPC), (Hamamatsu, Japan) [19], as a less expensive and easier to handle solution for detection. The MPPC has a spectral response covering the 320 to 900 nm spectral range, with a peaking at 440 nm, a dark count equivalent to 500 kcps, and a photon detection efficiency of 45%, at peak wavelength. The data acquisition form the MPPC is performed with the software provided by the manufacturer. The detector was used in the “pulsed count” mode. For these cases, the gate time was from 1 to 100 ms, whereas the threshold level was set from 1.5 to 6.5 p.e. The spectrometer was used to visualize the spectral response of the sensor, as far as the C10507-11-100U photon counter is not able to provide information of the spectral content of the radioluminescent signal.

## 3. Results and Discussion

Two types of sensor exposures to X-ray were used during these studies: one along the longitudinal axis of the cylinder defining the sensor tip, in 0.5 mm steps, and the other, frontal on the tip cap, masking the optical fiber core end, coaxial with the optical fiber core. For each sensor, the longitudinal scanning was done for four positions, as the sensor was rotated in increments of 90° after each complete longitudinal scan. The frontal scan was employed for both X-ray fluorescence and radioluminescence tests, while the longitudinal scan was performed for radioluminescence assessment only. In the first case, the X-ray beam was focused to 2 mm, whereas for the longitudinal scan the beam diameter was 1mm. In addition, in the frontal test, the surface distribution of the sensor responsivity was evaluated, by scanning the sensor end with a focused X-ray beam of 1 mm.

The X-ray fluorescence provides information on the concentration of the active material, as the sensor response to X-ray depends on it. Combining the X-ray fluorescence with the radioluminescence response can be derived the efficiency to generate the optical signal and its coupling efficiency to the optical fiber. Longitudinal scanning for the radioluminescence detection along the optical fiber offer information on the efficiency to couple the optical radiation to the optical fiber on its sides and helps the evaluation of the concentricity exiting between the optical fiber core and the sensor tip.

Measurements performed on the sensor tip end (frontal) make possible the assessment of the sensors response dynamic range, linearity, and reproducibility, as function of the X-ray source parameters.

Comparing the results of the radioluminescence tests with those derived from X-ray tomography, the sensor design can be investigated. In the mean time these examination provides information on sensor's spatial resolution.

The measurements carried out with the C10507-11-100U MPPC coupled to the optical fiber sensor makes possible the setting of the operating conditions for such an X-ray detector for different parameters of the excitation source.

### 3.1. X-Ray Fluorescence and Radioluminescence Measurements at Sensors Tip End

**Figure 1.**
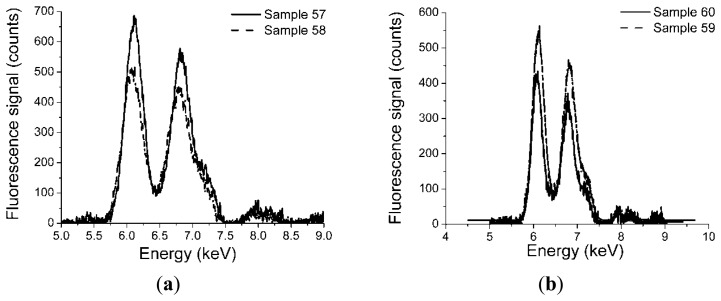
Comparison of the elemental composition of two sets of sensors pairs: (**a**) sensors 57 and 58; (**b**) sensors 59 and 60, obtained through X-ray fluorescence at the tip ends.

As six sensors were produced using similar glue-phosphor mixture preparation methods, we were interested in examining the reproducibility of this process. For this purpose, X-ray fluorescence tests were done in a frontal irradiation geometry, for sensors pairs 57/58, and 59/60 to check the elemental composition, by exposing the sensor tip end to a 2 mm diameter X-ray beam in its central region, with an angle of incidence of 22.5 degrees and the angle of detection of 22.5 degrees. The X-ray source driving voltage was V = 40 kV, at a driving current of I = 90 μA. These values were kept constant during the tests. Figure 1 shows the fluorescence response of the characterized sensors. It can be noticed that the elemental composition does not differ more than 10%. The difference is not significant considering the manual manufacturing process involved. This is a factor which has to be accounted when comparing the responsivity of different sensors.

X-ray fluorescence provides information on several peaks associated to the present ingredients. In this case, spectra of Gd and Tb Lα lines, Gd and Tb Lβ lines were obtained. In order to estimate the contribution of the two constituents we considered the Gd Lβ, Tb Lβ lines, as the α line of the two elements overlap and it is difficult to distinguish them one against the other.

**Figure 2.**
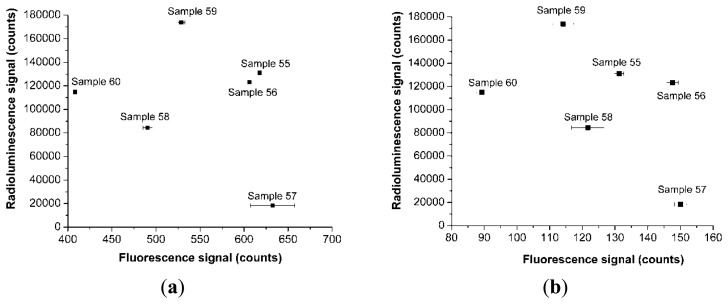
The plot of the radioluminescence signal generated by the sensor versus the amplitude of the X-ray fluorescence signal associated to the peak of Gd Lβ (**a**), Tb Lβ (**b**). The operating conditions of the X-ray source are V = 40 kV, I = 90 μA.

The next step of our research was focused on the evaluation of these constituents contribution to the detected optical signal. For this reason, we plotted the optical response signal (radioluminescence) detected by the QE65000 spectrometer coupled to the other end of the sensor optical fiber versus the amplitude of the fluorescence signal picked up by the Silicon Drift Detector. The measurements were done simultaneously, under the same operating conditions of the X-ray source. Examples of these results are given in Figure 2.

The Gd_2_O_2_S:Tb radioluminescence emission spectrum has four peaks, the major one centered at 542 nm. The PMMA optical fiber exhibits two spectral bands with an increased transmission from 350 to 540 nm, and from 580 to 640 nm.

Comparing the two spectra, the emission and the transmission ones, a reasonable match can be noticed, which offer a good efficiency of the sensors to X-ray detection.

The linearity of sensors response as function of the driving conditions of the X-ray source was measured for all the four peaks of the spectrum.

**Figure 3.**
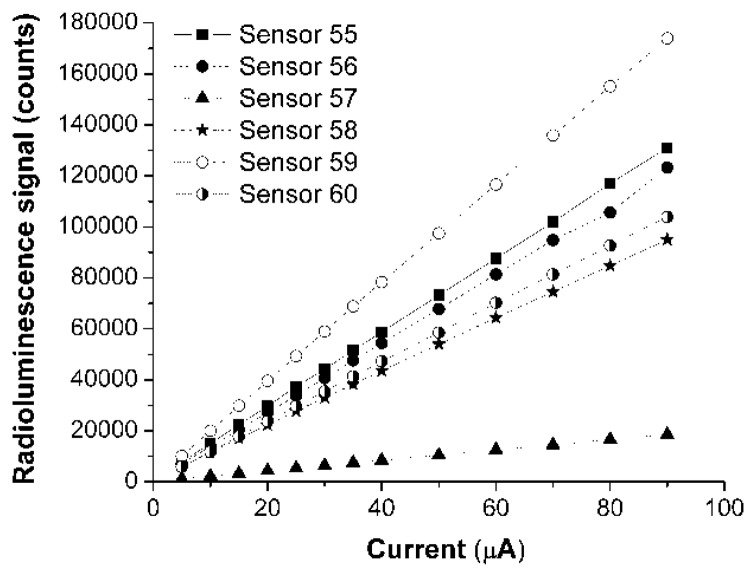
The responsivity of sensors at λ = 542 nm, as function of the X-ray source driving current, for the driving voltage V = 40 kVp.

**Figure 4.**
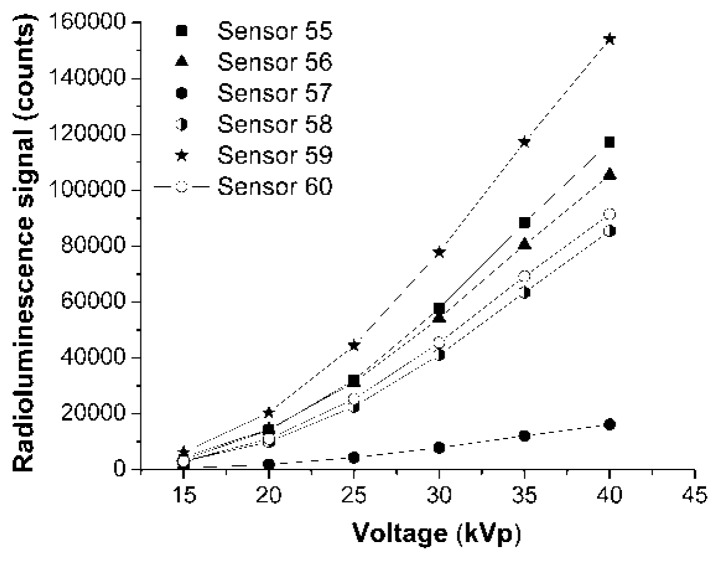
The responsivity of sensors at λ = 542 nm, as function of the X-ray source driving voltage, for the driving current I = 80 μA.

A comparison of the responsivity of the four sensors based on the same glue-phosphor mixture, being developed with different techniques is done in Figures 3 and 4. The variable is either the driving current or the driving voltage of the X-ray source. In Figures 3 and 4 are represented the response associated to the main emission peak (λ = 542 nm), integrated over its associated spectral interval.

**Figure 5.**
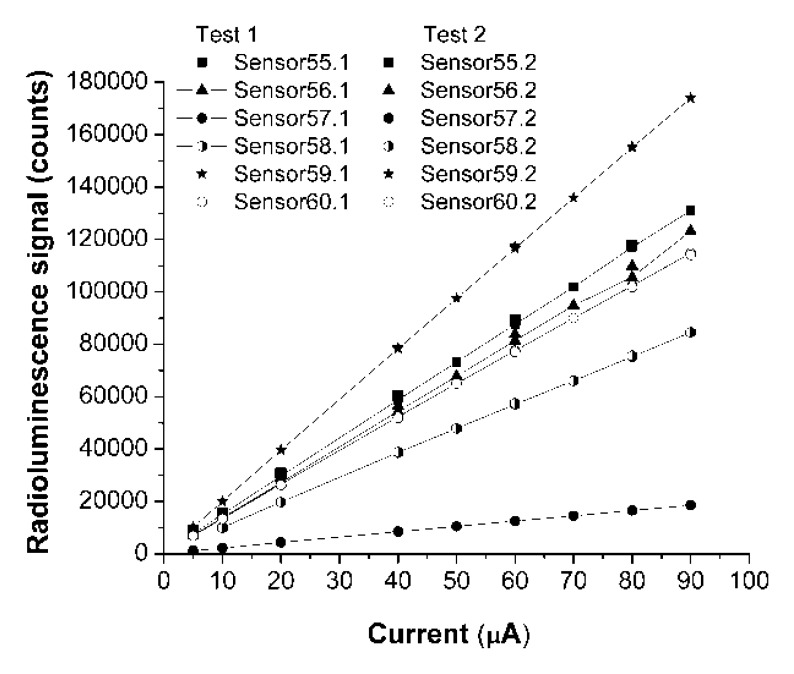
The hysteresis tests for sensors.

For a fixed value of the X-ray source driving voltage two tests were run for each sensor to evaluate the repeatability of their response. Figure 5 indicates the lack of hysteresis for the sensors response, in the case sensors 55–60.

### 3.2. X-Ray Radioluminescence Measurement along Sensors Longitudinal Axis

**Figure 6.**
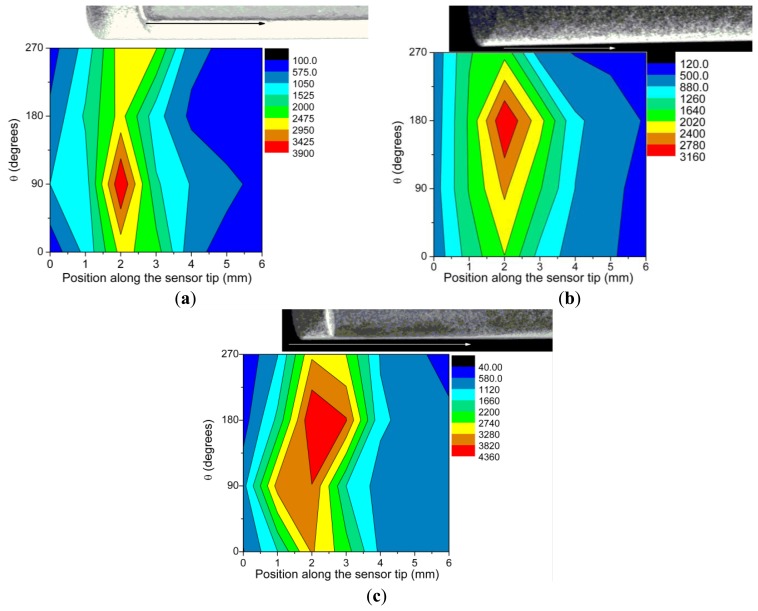
The map of sensors responsivity (**a**) 55; (**b**) 56; (**c**) 59, along the tip longitudinal axis, for four positions as the sensor was rotated with respect to its axis.

In order to evaluate the coupling efficiency of the generated optical radiation along the optical fiber over which the sensing tip is located radioluminescence measurements were performed by scanning the sensor tip along a line parallel to the optical fiber axis. The tests were done for four positions of the tip as it was rotated in 90° steps with respect to its geometrical axis. These measurements provide information on the way the optical radiation generated along the tip length is coupled to the optical fiber core and, in addition, offer some insides on the eccentricity of the phosphorescent tip as compared to the optical fiber core. Figure 6 reproduces the mapping of the responsivity for the above mentioned cylindrical geometry of irradiation. The X-ray operating parameters were driving current I = 90 μA and driving voltage V = 40 kVp, for all four sensors. In each case, over the responsivity map was superposed a longitudinal cross section of the respective sensor X-ray tomography to help the location of the detected signal against the X-ray focused beam scanning position. In the image the tip cap and the end of the optical fiber core can be observed. The scanning runs from left to right, from tip end toward the optical fiber. As mentioned before the X-ray beam diameter was focused down to 1 mm, and the scanning was performed in 0.5 mm steps.

### 3.3. The Study of Reflector Influence on the Responsivity

**Figure 7.**
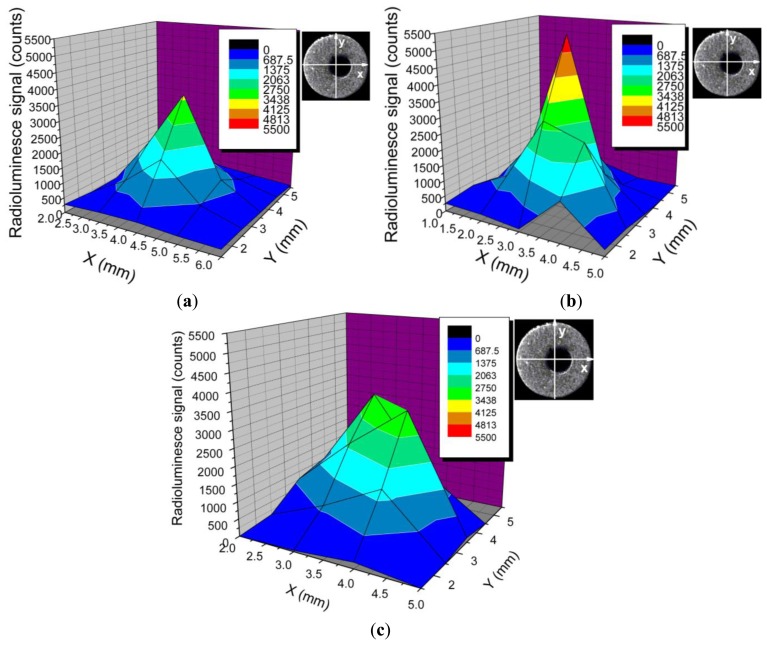
The effect of the external reflector on the efficiency of generated optical signal coupling to the optical fiber core: (**a**) sensor 56 without a reflector; (**b**) the same sensor with an Al foil reflector; (**c**) the same sensor coated with TiO_2_.

As we noticed, the most efficient region for the coupling of the generated optical radiation to the fiber core is located near the tip end. In order to improve the sensor response, we performed some measurements of sensors responsivity to X-ray by covering the tip with two reflecting materials: an Al thin foil and a TiO_2_ paste. The improvements obtained by using these reflectors are illustrated in Figure 7, where the radioluminescence signal is plotted as the sensor cap surface is scanned with a focused X-ray beam. As can be observed, the addition of a reflector increases the sensors light collecting efficiency. For an easier interpretation of the results, a transversal cross section through each sensor tomographic image was added and a cross section was done at the bottom end of sensor's tip.

**Figure 8.**
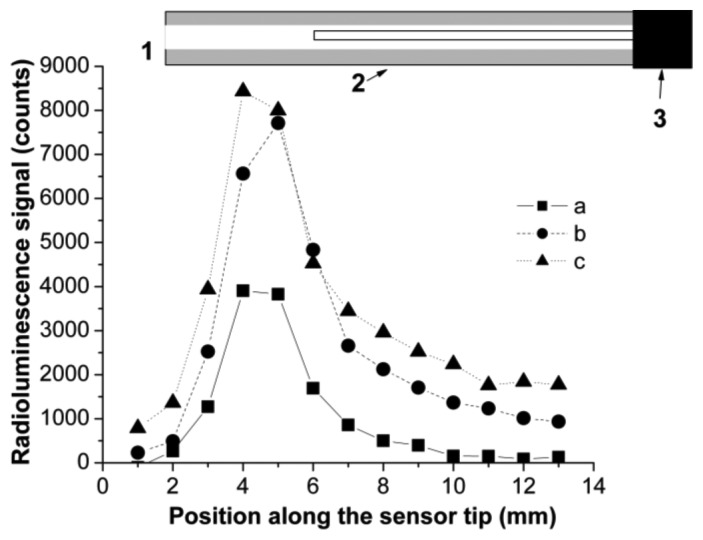
The efficiency of optical signal collection with two reflectors and without them for sensors 59: (**a**) no reflector; (**b**) Al foil; (**c**) TiO_2_ paste. Sensor elements are identified as follows: (1) sensor tip; (2) reflector; (3) PMMA optical fiber core.

The evaluation of reflectors efficiency for light collection in the guiding optical fiber was done by scanning the sensor 56 along its longitudinal axis and measuring the radioluminescence signal at the other end of the optical fiber (Figure 8).

### 3.4. Sensor Operation with the MPPC Unit

**Figure 9.**
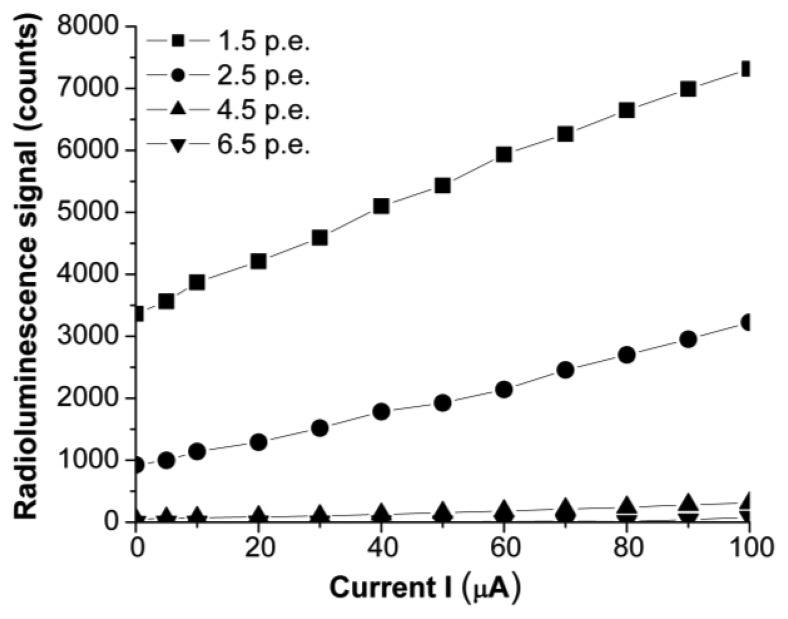
The change at the MPPC output as function of the X-ray driving current, at 20 kVp driving voltage, for four threshold levels.

The use of a sensitive optical fiber spectrometer with the proposed X-ray sensor could be an expensive solution. For this reason, we tested its operation in conjunction with a Hamamatsu MPPC type C10507-11, used in the photon counting mode. In addition, the second approach is easier to handle for field applications. Section II details the working parameters we set for this electronic unit. The response of the entire sensor system (X-ray detector and electronics) for different parameters of the X-ray source is given in Figure 9, for sensor 59.

### 3.5. Discussion

The investigations reported in this paper are focused on the design characteristics and operating parameters of a set of six extrinsic optical fiber sensors intended for X-ray monitoring and detection in low dose application. The sensors were manufactured with the same technology; the only differences being their dimensions and the way the epoxy-phosphor mixture are fixed at the end of a PMMA optical fiber.

X-ray fluorescence studies at 22.5 degree incidence on the sensor tip cap made possible the detection of the two major constituents of the phosphor: Gd and Tb. The concentration of Gd and Tb was evaluated from the Lβ lines. Based on these concentrations which are proportional to the fluorescence signal we derived the relation between the concentrations and the efficiency of the radioluminescence generation. This relation plotted in Figure 2 shows that the overall responsivity of the sensor depends not only on the Gd or Tb concentration but also on the technology used, as this one influences the coupling efficiency of the optical radiation to the guiding optical fiber. These results are supported by the plots in Figure 6 which indicate a higher value of the radioluminescence signal for sensor 59 (heat shrink technique) as compared to sensors 55 and 56 (plastic cylinder). This is also confirmed also by data from Figure 2.

The X-Y scanning along the tip length with a focused X-ray beam indicated that most of the detected optical radiation is generated and coupled in the proximity of the tip end. So, a length of the sensor tip higher than 5 mm has little contribution to the optical response, meaning that lateral coupling of the optical signal to the optical fiber is quite poor. On the other hand, this scanning run in a cylindrical geometry supports the finding from X-ray tomography providing an inside view on the eccentricity existing between the tip and the supporting optical fiber core. Such differences are important when the sensor is used in relation to focused X-ray beams, this eccentricity depredating the spatial uniformity of the response to X-ray. For applications where the sensor is “emerged” into an X-ray environment this manufacturing drawback has a lower impact on the sensor response. The collection of the radioluminescence signal can be improved by coating the tip with an Al foil or a reflecting painting, as more radiation generated along the tip length is directed to the fiber core. The coating of the sensor tip with a TiO_2_ layer seems to be more efficient than the use of an Al foil, as the foil is only rolled over the tip, where the paint has a better contact with the tip surface.

The use of the C10507-11 MPPC can be a cheaper and easy to handle solution to pick up and convert the optical signal from the sensor to a processing unit. The parameters set for the MPPC have to be selected according to the operating conditions of the X-ray source in order to have a linear response and a good sensitivity. A high value of the X-ray source driving voltage can produce too energetic photons which can saturate the C10507-11 unit, for low values of the detecting threshold.

## 4. Conclusions and Future Work

A new type of extrinsic optical fiber sensor for low X-ray doses was evaluated. This investigation was centered on evaluation tests run at micro scale, with focused X-ray beams. The sensitivity of all sensors has a linear dependence on the driving current of the X-ray source. A higher value of this current involves more incident photon and produces more optical photons, the conversion mechanism being a linear one within the selected current range and at a fix driving voltage.

The responsivity of the studied sensors as function of the driving voltage, for a fixed driving current, indicates a non-linear relation between the driving voltage and the detected optical signal. The non-linear dependence is supposed to be due to the variation of the penetration depth of X-ray photons in the shield used to cover the sensor tip in order to minimize the contribution of the ambient light to the detected signal. The tested sensors indicate no hysteresis with respect to the operating conditions of the excitation source. A very good reproducibility of the reading was obtained.

Comparing the spectrum of the radioluminescence signal with the transmission spectrum of the PMMA optical fiber we notice that the efficiency of the sensor response to X-ray can be further improved by selecting a phosphor having an emission maximum shifted towards 450 nm.

In the future our research will be directed towards the increase of sensors responsivity to X-ray by designing new geometries to couple the radioluminescence signal to the optical fiber core, and on the other side, to improve the transmission of the optical guide.
